# Using natural language processing and machine learning to classify health literacy from secure messages: The ECLIPPSE study

**DOI:** 10.1371/journal.pone.0212488

**Published:** 2019-02-22

**Authors:** Renu Balyan, Scott A. Crossley, William Brown, Andrew J. Karter, Danielle S. McNamara, Jennifer Y. Liu, Courtney R. Lyles, Dean Schillinger

**Affiliations:** 1 Ira A. Fulton School of Engineering, Arizona State University, Mesa, Arizona, United States of America; 2 Department of Applied Linguistics/ESL, College of Arts and Sciences, Georgia State University, Atlanta, GA, United States of America; 3 UCSF Center for Vulnerable Populations, Department of Medicine, University of California, San Francisco, California, United States of America; 4 Division of Research, Kaiser Permanente Northern California, Oakland, California, United States of America; 5 Psychology Department, Arizona State University, Tempe, Arizona, United States of America; 6 Zuckerberg San Francisco General Hospital and Trauma Center, San Francisco, California, United States of America; STL UMR8163 CNRS, FRANCE

## Abstract

Limited health literacy is a barrier to optimal healthcare delivery and outcomes. Current measures requiring patients to self-report limitations are time-consuming and may be considered intrusive by some. This makes widespread classification of patient health literacy challenging. The objective of this study was to develop and validate “literacy profiles” as automated indicators of patients’ health literacy to facilitate a non-intrusive, economic and more comprehensive characterization of health literacy among a health care delivery system’s membership. To this end, three literacy profiles were generated based on natural language processing (combining computational linguistics and machine learning) using a sample of 283,216 secure messages sent from 6,941 patients to their primary care physicians. All patients were participants in Kaiser Permanente Northern California’s DISTANCE Study. Performance of the three literacy profiles were compared against a gold standard of patient self-reported health literacy. Associations were analyzed between each literacy profile and patient demographics, health outcomes and healthcare utilization. T-tests were used for numeric data such as A1C, Charlson comorbidity index and healthcare utilization rates, and chi-square tests for categorical data such as sex, race, poor adherence and severe hypoglycemia. Literacy profiles varied in their test characteristics, with C-statistics ranging from 0.61–0.74. Relations between literacy profiles and health outcomes revealed patterns consistent with previous health literacy research: patients identified via literacy profiles indicative of limited health literacy: (a) were older and more likely of minority status; (b) had poorer medication adherence and glycemic control; and (c) exhibited higher rates of hypoglycemia, comorbidities and healthcare utilization. This represents the first successful attempt to employ natural language processing to estimate health literacy. Literacy profiles can offer an automated and economical way to identify patients with limited health literacy and greater vulnerability to poor health outcomes.

## Background and significance

An estimated 30.3 million people in the U.S. had diabetes mellitus (DM) in 2015, according to the Centers for Disease Control and Prevention (2017). Like most chronic conditions, DM self-management can be complex and requires frequent communication between patients and their healthcare providers. Health literacy (HL) is generally defined as a patient’s ability to obtain, process, comprehend, communicate and act on basic health information [1, 2]. DM patients with limited HL have a higher risk of poor health outcomes, including worse blood sugar control, higher complication rates [3] and a greater incidence of hypoglycemia [4, 5]. Poor communication and sub-optimal adherence to medication may explain some of these disparities [6, 7]. Limited HL contributes to preventable suffering, more rapid decline in physical function [8] and related excess healthcare costs.

Online patient portals embedded within electronic health records (EHRs) are now being used widely to bridge in-person encounters and provide support between visits by allowing patients and providers to communicate via secure messages (SMs). Kaiser Permanente Northern California (KPNC) has a well-developed and mature patient portal, kp.org. Previous research suggests that patients who access such portals are more likely to have better (a) healthcare utilization [9], (b) medication adherence [10–11] and (c) glycemic (blood sugar) control [12–13]. Among DM patients, better ratings of physician communication are associated with greater SM usage [14]. The reach and effectiveness of online communication is affected by patients’ HL. While limited HL may complicate access to patient portals and impacts patients’ evaluation of online health information [15], diabetes patients with limited HL are increasingly using patient portals. In 2014, 68% of KPNC DM patients with limited HL and 84% with adequate HL accessed the portal [DISTANCE Study, unpublished data]. Overall, 46% used SM in 2014, compared to 30% in 2009. Those with limited HL are rapidly gaining ground, showing a 65% increase in a 5-year period compared to a 41% increase for those with adequate HL. The greatest gains have been among Latinos and African Americans, suggesting that social differences in utilization are narrowing.

No research has harnessed SMs to identify patients with limited HL. Developing scalable tools to identify limited HL without the burden of primary data collection would be an efficient way to enable tailored provider communication and related interventions. Goals of the ECLIPPSE study (Employing Computational Linguistics to Improve Patient-Provider Secure Email exchanges) are to (a) develop patient literacy profiles (LPs) using natural language processing (NLP) to classify HL (limited vs. adequate) in a large sample of SMs from diabetes patients, and (b) assess whether LPs are associated with patient demographics and health outcomes. We hypothesize that patients’ language constructs in portal communications can be harnessed to identify patients with limited health literacy.

### Related research

Prior research in medical domains has benefitted from the use of NLP combining computational linguistics with machine learning (ML). Such studies include representation of clinical narratives, assessing medical articles’ readability, text quality, and developing semantic lexicons for medical language processing [16–23]. Some of the commonly used NLP tools and techniques employed are Apache clinical text analysis and knowledge extraction system (cTAKES) [24], the clinical language annotation, modeling, and processing tool (CLAMP) [25], the medical language extraction and encoding system (MedLee) [26] and the Kawasaki disease-NLP (KD-NLP) [27] tool. Additionally, tools like the KnowledgeMap (KM) concept identifier can extract concepts represented in medical educational texts [28] while the MetaMap [29] system provides links from biomedical texts to concepts in the unified medical language system (UMLS) Metathesaurus [30]. Other NLP applications include The Pharmacogenomics/ Pharmacogenetics Knowledge Base (PharmGKB) [31–32], LinKBase [33], medical ontologies, and lexicons such as BioLexicon [34], UMLS [30] and medical WordNet (WMN) [35].

With the increase in NLP tools, the readability of medical texts has also become an important research area [36–42]. Some of the most commonly used tools for measuring readability of medical texts are Flesch-Kincaid Grade level (FKGL) [43], SMOG [44–45], Gunning-Fog Index (GFI) [46] and suitability assessment of materials (SAM) [47]. Despite their popularity, these classic readability formulas have faced criticism from scholars because they ignore critical aspects of text that contribute to comprehension difficulty [48–49, 39–40, 42]. For instance, Kim et al. [39] developed a readability-scoring algorithm for evaluating medical text using NLP techniques (e.g., text length features, syntactic and semantic features, and concept familiarity scores). They compared their algorithm to classic readability formulas and found that their metric was a viable alternative. Wu et al. [40] extended Kim’s work to a larger corpus of medical documents and found that classic readability formulas may not produce meaningful scores for medical texts. More recently, Zheng and Yu [42] used a supervised ML approach to assess readability of medical documents using text features and word embeddings. Their approach achieved higher concordance with human annotators than the FKGL. Related work in languages other than English have reported similar results, including work by Grigonyté et al. [50] for EHRs written in Swedish and Venturi et al. [51] for informed consent forms in written Italian.

Despite challenges unique to bio-text mining, NLP and ML tools and techniques are also gaining importance. NLP and ML are now used in medical text analyses for terminology processing: extraction of named entities (TerMine) [52], information extraction (MEDLINE information extraction-MEDIE), semantic information retrieval (KLEIO) [53], association mining (FACTA) [54], and linking texts to pathways (PathText) [55].

These tools have been used for clinical analyses and not to measure HL. The few formulas used in HL studies (e.g., Flesch-Kincaid and SMOG) depend on surface-level features that center on shallow lexical and sentential indices. Despite the increasing use of NLP and ML techniques in health domains, to our knowledge, no study has utilized these techniques to estimate the HL of patients. Kim and Xie [56] carried out a literature survey to identify online health services used by people with limited HL and concluded that there is a need for new HL screening tools. Healthcare delivery systems are recognizing the importance of identifying the significant subset of patients who have limited HL. Measuring HL, however, requires the use of individual interviews or questionnaires, rendering the process time-consuming and challenging, especially for larger patient populations. An automated LP based on NLP would provide a more efficient means to identify large numbers of patients with limited HL. ECLIPPSE set out to develop an automated LP prototype that can (a) identify patients with potential HL limitations in an automated way, (b) determine whether the measures are predictive of self-reported HL and are associated with socio-demographic characteristics and health outcomes, and (c) deliver feedback to clinicians about the HL skills of patients so that clinicians can modify their language to make SMs more readable and actionable, thereby improving communication. The current paper attempts to accomplish the first two objectives using LP models created generated from NLP and ML techniques.

## Materials and methods

### Data source and participants

Data for this study were extracted from the KPNC Diabetes Registry (N~320,000, as of 01/01/2017). Our sampling frame includes >1 million SMs generated by >150,000 ethnically diverse DM patients and >9,000 clinicians from KPNC, a fully integrated health care delivery system. We identified the subset of these patients who completed a 2005–2007 survey entitled the Diabetes Study of Northern California (DISTANCE), including providing self-reported HL (N = 14,357) [57–59]. DISTANCE involved a survey of DM patients receiving care from KPNC, oversampling minority sub-groups to assess the role of socio-demographic factors on quality of care. The variables in DISTANCE were collected from questionnaires completed via telephone, on-line, or paper and pencil (62% response rate).

We extracted all the SMs (N = 1,050,577) exchanged between a patient and all clinicians from KPNC’s patient portal between 01/01/2006 and 12/31/2015. We then identified those SMs that a patient sent to his or her primary care physician(s). Those patients who did not have matching DISTANCE survey data were removed. We then removed all SMs written in a language other than English and all SMs identified as written by proxies (i.e., SMs written for the patient by caregivers) [60]. The length of SMs varied between 1 word and 16,469 words, and average length of the SMs was 2,058.95 words. The range of number of SMs sent by a patient who participated in the DISTANCE survey to their physician(s) varied between 2 and 205, and the average number of SMs sent were 39.88. All SMs from each patient were collated into a single file from which we could extract the linguistic features. Patients whose aggregated SMs lacked sufficient words (<50 words) to provide linguistic coverage were removed. Our 50-word threshold was based on previous NLP text analyses in learning analytics domains [61–62]. The final cleaned data consisted of 6,941 patients and 283,216 SMs. The linguistic features derived from these SM were used to predict HL based on self-reported HL scores obtained from survey data. The ECLIPPSE Study was approved by the KPNC Institutional Review Board (IRB). Because these analyses involved secondary data only and because these data are housed on a password-protected secure server that can only be accessed by KPNC-approved and ethics–certified researchers, and because analyses predominantly employed computational techniques which yielded a quantitative measure of linguistic complexity, the KPNC IRB waived the requirement for patient consent.

### Natural language processing tools

In order to predict the patients’ self-reported HL scores, linguistic features were derived from the patients’ SMs to their primary care physicians. For this study, we used a number of NLP tools to select linguistic indices that measure different language aspects, such as text level information (e.g. number of words in the text, token type ratio), lexical sophistication, syntactic complexity, and text cohesion (e.g. connectives, word overlap). The NLP tools used included the Tool for the Automatic Assessment of Lexical Sophistication (TAALES) [63–64], the Tool for the Automatic Analysis of Cohesion (TAACO) [65], the Tool for the Automatic Assessment of Syntactic Sophistication and Complexity (TAASSC) [66–67], the SEntiment ANalysis and Cognition Engine (SÉANCE) [68], and the Writing Assessment Tool (WAT) [69–70]. These NLP tools in turn used a Stanford Parser [71], British National Corpus (BNC) [72], MRC psycholinguistic database [73], CELEX word frequency database [74] and Wordnet [75]. In addition, we used medical corpora such as HIMERA [76], i2b2 [77–80] unannotated data released during 2006–2014 to generate the frequencies of all medical terms used in these corpora (data available at https://www.i2b2.org/NLP/DataSets/Main.php). The features used in the models were extracted only if they were normally distributed, not multi-collinear and demonstrated at least a small effect size. These NLP tools were previously developed specifically to measure language features related to text complexity, readability and cohesion each of which is associated with literacy. However, they were not developed specifically for e-mail communication or for medical or clinical corpora. A brief description of these tools follows.

#### Tool for the automatic assessment of lexical sophistication (TAALES)

TAALES [63–64], incorporates over 200 indices related to lexical information. The indices include number of types and tokens for both words and n-grams, lexical frequency, lexical range (i.e., the number of documents in which a reference item occurs), word information measures (e.g., concreteness, familiarity, meaningfulness), psycholinguistic features (e.g., word neighborhood effects, word name and response latencies), word association strengths, and academic words and phrases.

#### Tool for the automatic analysis of cohesion (TAACO)

TAACO [65] incorporates over 200 classic and more recently developed indices related to text cohesion. For a number of indices, the tool incorporates a part of speech (POS) tagger and synonym sets from the WordNet lexical database [75]. Specifically, TAACO calculates type token ratio (TTR) indices, sentence and paragraph overlap indices that assess local cohesion and global cohesion at the word and semantic level, and incidence of connectives and conjunctions.

#### Tool for the automatic assessment of syntactic sophistication and complexity (TAASSC)

TAASSC [66–67] measures large clausal and phrasal indices of syntactic complexity and usage-based frequency/contingency indices of syntactic sophistication. TAASSC includes 14 indices measured by Lu’s Syntactic Complexity Analyzer (SCA) [81], 31 fine-grained indices or clausal complexity, 132 fine-grained indices of phrasal complexity, and 190 usage-based indices of syntactic sophistication.

#### Sentiment analysis and cognition engine (SÉANCE)

SEANCE [68] is a sentiment analysis tool that relies on a number of pre-existing sentiment, social positioning, and cognition dictionaries. SEANCE provides a negation feature (i.e., a contextual valence shifter) and includes a part of speech (POS) tagger for many indices.

#### Writing assessment tool (WAT)

WAT [69–70] was developed specifically to assess writing quality. As such, it includes a number of writing specific indices related to text structure (text length, sentence and paragraph length), cohesion (e.g., local, global, and situational cohesion), lexical sophistication (e.g., word frequency, hypernymy, meaningfulness, age of acquisition), keyword use, part of speech tags (e.g., nouns and verbs), syntactic complexity (e.g., number of constituents in a clause), and rhetorical features (e.g., hedges and downtoners).

### Variables

#### Primary predictors: The linguistic features and resultant literacy profiles (LPs)

We analyzed the patients’ SM to derive a set of 185 linguistic features calculated by the tools above to generate LPs and explore the extent to which each predicts self-reported HL. The linguistic aspects chosen for this study have previously been shown to predict literacy levels in non-clinical corpora [82–83]. A sample of the employed linguistic indices, their descriptions and hypothesized relation to HL are briefly described in Table 1.

**Table 1:** Selected NLP indices and relation to health literacy (HL) scores.

Linguistic Index	Description	Relation to Health Literacy (HL)
Concreteness	The degree to which a word is concrete or imageable vs. abstract (e.g., table vs. love)	Less concrete words in high HL patient writing
Lexical diversity	Lexical diversity refers to the variety of words used in a text. It is usually measured using type–token ratios (TTR), which is related to text length	More lexical diversity (i.e., more diverse words) in high HL patient writing
Present tense	Incidence of present tense	Less use of present tense in high HL patient writing
Determiners	Incidence of determiners (e.g., *a*, *the*)	More determiners in high HL patient writing
Adjectives	Incidence of adjectives	More adjectives in high HL patient writing
Function words	Incidence of function words such as prepositions, pronouns etc.	More function words in high HL patient writing

#### Dependent variable(s): Self-reported health literacy

As a gold standard, we used combinations of self-reported HL items from the DISTANCE survey to compute three dependent variable versions of predicted self-reported HL. The survey included the following HL measures: self-reported “confidence in filling out medical forms” (HLCONF), “problems in understanding written medical information” (HLPROB), frequency of “needing help in reading and understanding health materials” (HLHELP); and an original item: “problems understanding prescription labels” (HLLABELS) [S1 Table]. The first three items have previously been validated [84]. Patient responses were collected using a 5-point Likert scale in which responses of 1 referred to “Always” and a 5 to “Never.” For our analyses, we combined these items to create different self-reported variables to compare the performance of the linguistic features against different computations of self-reported HL (i.e., combined HL [HLCOMB], trinary summed HL [HLSUMTri], and average HL [HLAVG]; see S1 Table for definitions and computation of these variables).

HLCOMB considers binary forms of three self-reported HL measures (HLPROB2, HLCONF2, and HLHELP2; a ‘zero’ score indicates that a patient reports no HL limitations and a ‘one’ that a patient reports limited HL on any one of the three items). HLSUMTri is a trinary variable computed by summing the Likert scale values obtained for HLPROB, HLCONF, and HLHELP. The HLSUMTri variable had three possible values ranging between 0 and 2. Zero (0) indicates a patient with limited HL, whereas one (1) and two (2) represent a patient with marginal and adequate HL, respectively. The HLAVG scores were computed by taking the mean of HLPROB, HLHELP, HLCONF, and HLLABELS (S1 Table).

#### Additional dependent variable(s): Socio-demographic characteristics and health outcomes

The average age of our study population at the time of the DISTANCE study was 56.8 (±10); 54.3% were male and 32.2% were white. Using data derived from the EHR, we examined medication adherence based on continuous medication gaps (CMG) [85–86], a validated adherence measure of percent time with insufficient medication supply; hypoglycemia (a side effect of DM treatment, which has been previously linked to limited health literacy [4]; Hemoglobin A1c (an integrated measure of blood sugar control); and Charlson index [87–88] (a measure of comorbidity and illness severity; we used the Deyo version of the Charlson comorbidity index) [89]. We considered patients to have poor adherence if CMG>20% [90]. A1c was the most recent value collected after the first SM sent since DISTANCE survey completion, and CMG, severe hypoglycemia and Charlson index were measured the year before the first SM was sent. The occurrence of any hypoglycemia-related ED visit or hospitalization was based on a validated algorithm [91] (any of the following ICD-9 codes: 251.0, 251.1, 251.2, 962.3, or 250.8, without concurrent 259.8, 272.7, 681.XX, 682.XX, 686.9X, 707.1–707.9, 709.3 730.0–730.2, or 731.8 codes). Another set of analysis was conducted for health service utilization, using outpatient clinic visits, emergency room encounters and hospitalizations.

### Statistical analysis

Analyses were conducted to develop LPs using several supervised ML algorithms [92–96]. We examined links between three summed self-reported HL variables (HLCOMB, HLSUMTri, and HLAVG) and the 185 linguistic predictor variables extracted using the linguistic tools. To perform binary classification, we categorized the summed self-reported HL scores into discrete levels (limited vs. adequate HL). We trained Weka (version 3.8.1) and R (version 3.3.2) implementations for the ML models, including linear discriminant analysis (LDA), support vector machines (SVM), naïve Bayes, random forests, and artificial neural networks. These algorithms are some of the simplest and the most commonly used algorithms for classification problems. We used 10-fold cross validation approach on 70% of the data for fine-tuning the parameters and validation of the model. The performance of the model was tested and reported on the held-out 30% data. In all cases, linguistic features were used to predict the discrete HL levels. Several metrics such as accuracy, sensitivity, specificity, positive and negative predictive values (PPV and NPV), and C-statistic (area under the receiver operator characteristic (ROC) curves) were used as measures of model performance using a split sample approach. The resulting LPs were subsequently validated against self-reported HL items and socio-demographic variables previously collected from the patients via in the DISTANCE survey [58], and the HL-sensitive health outcomes obtained from administrative data from the EHR, described above. We discuss the results of the three models that performed the best for each of the dependent variables.

To examine whether the ML approaches resulted in patterns similar to those reported in prior literature on self-reported and directly measured HL, we examined bivariate associations between each of the LP models and socio-demographic, health outcome and healthcare utilization variables using a two-sided p-value at the 0.05 level of significance. Categorical variables such as sex, race, poor adherence [90] and severe hypoglycemia were analyzed using chi-square analysis. Mean comparisons were conducted using t-tests for A1c, Charlson (comorbidity) index [87], healthcare utilization rates.

## Results

### Aggregated health literacy measures

The first analysis to create an LP model used HLCOMB as the dependent variable. The data for HLCOMB were distributed uniformly, with 3,229 patients having adequate HL (or no HL limitations), and 3,712 limited HL. The LDA model performed the best for this version of the LP, achieving an accuracy of 60.55% and a C-statistic of 0.63 for the test data (Table 2; bold entries indicate the highest value for a given metric within an LP).

**Table 2:** Classification metric statistics of models for different self-reported literacy profiles (Positive class: Adequate HL).

ML Algorithm for Literacy Profiles	Literacy Profile (Dependent Variable)	Accuracy	C-statistic	Sensitivity	Specificity	Positive Predictive Value (PPV)	Negative Predictive Value (NPV)	# of Predicted limited vs adequate HL*
LDA	HLCOMB	60.55	0.63	56.10	64.42	57.83	62.78	1142 / 939
LDA	HLSUMTri	**63.58**	0.61	39.32	**79.32**	55.23	**66.82**	1498 / 583
SVM	HLAVG	62.52	**0.74**	**75.49**	47.11	**62.91**	61.79	725 / 1356

* The numbers are a function of sample size for test set only

The second analysis considered HLSUMTri as the dependent variable to create an LP. Since the HLSUMTri variable had three possible values (classes), we used multiclass classification. The accuracy of the models was lower and ranged between 50.67% and 54.23%. SVM achieved the highest accuracy. However, SVM classified all instances as marginal or adequate HL. To explore if these algorithms performed using binary classification, we combined the inadequate (0) and marginal (1) HL instances and re-classified these as limited (0+1) HL, while the adequate (2) HL cases were retained. In binary classification, the LDA model performed the best, and the results were better than the multiclass classification results. The LDA model achieved an accuracy of 63.58% and a C-statistic of 0.61. However, the C-statistic was lower than the LDA model of the LP trained using HLCOMB, as was its sensitivity (39.32% vs. 56.10%, Table 2).

For the third analysis, we considered the HLAVG scores as the dependent variable to create an LP. The data set included 3,173 limited HL and 3,768 adequate HL instances. Accuracy and c-statistic for this SVM model were 62.52% and 0.74 respectively. While the specificity was lower, it achieved the greatest balance in PPV and NPV (Table 2).

### Linguistic characteristics

The LP models generally showed that patients with predicted limited HL produced messages having fewer words, and those words were less sophisticated (i.e., more concrete) and demonstrated less lexical diversity (i.e., greater repetition of words). Additionally, patients with predicted limited HL produced more words that expressed negative affect (i.e., more words related to failure and fewer positive words). Lastly, predicted limited HL patients focused less on personal language, using a greater incidence of third person pronouns and fewer first person pronouns.

### Demographics

When applying the ML model-derived LPs to the validation dataset, we found patterns that matched previously observed relationships between patient demographic characteristics and HL. For example, patients identified by the LPs to have limited HL were 1–3 years older than high HL patients. In addition, 70.8–76.1% of the predicted limited HL patients were non-white, compared to 59.9–63.5% of adequate HL patients (Table 3), and 84.7–88.7% of patients with predicted limited HL had high school diplomas compared to 93.4–95% of patients with adequate HL.

**Table 3:** Demographics (Sex %, Race % and Age–Mean (SD)).

ML Algorithm for Literacy Profiles	Literacy Profile (Dependent Variable)	Sex—Men %			Race–White %		Age at Survey–Mean (SD)		P-value
		Limited HL	Adequate HL	P-value	Limited HL	Adequate HL	Limited HL	Adequate HL	
LDA	HLCOMB	54.9	53.7	0.32	25.5	40.0	57.91 (10.0)	55.53 (9.66)	< 0.001
LDA	HLSUMTri	55.8	53.6	0.08	29.2	40.1	57.34 (10.0)	55.43 (9.50)	< 0.001
SVM	HLAVG	53.6	56.2	0.06	23.9	36.5	58.88 (9.98)	55.74 (9.74)	< 0.001

### Health outcomes

To evaluate whether LPs were associated with health outcomes in the anticipated directions, we linked these modeled LP scores to outcomes previously found to be associated with measured HL. The results for medication adherence for LP models using HLCOMB and HLSUMTri lacked significance, whereas the model for HLAVG was statistically significant (Table 4). Patients with limited HL based on this LP were more likely to have poor medication adherence than high HL patients (24.5%-25.6% vs. 23.2%-23.4%). Patients predicted to have limited HL also had higher severe hypoglycemia rates in all the models, with SVM distinguishing the most. In sum, the SVM version of the LP HLAVG appeared to be the LP that performed best.

**Table 4:** Poor adherence and hypoglycemia (%).

ML Algorithm for Literacy Profiles	Literacy Profile (Dependent Variable)	Poor medication adherence (%)			Severe Hypoglycemia (%)		
		Limited HL	Adequate HL	P-value	Limited HL	Adequate HL	P-value
LDA	HLCOMB	24.9	23.3	0.143	4.0	2.0	< 0.001
LDA	HLSUMTri	24.5	23.2	0.296	3.5	2.1	< 0.001
SVM	HLAVG	25.6	23.4	0.047	5.1	2.0	< 0.001

Table 5 shows that patients predicted to have limited HL as measured by the LP HLAVG had poorer glycemic control. Patients with predicted limited HL also had higher prevalence of comorbid conditions compared to those with adequate HL. Again, the SVM version of the LP HLAVG appeared to be the LP that performed best.

**Table 5:** A1c and Charlson index—Mean (SD).

ML Algorithm for Literacy Profiles	Literacy Profile (Dependent Variable)	A1c			Charlson Index		
		Limited HL	Adequate HL	P-value	Limited HL	Adequate HL	P-value
LDA	HLCOMB	7.51 (1.56)	7.48 (1.50)	0.371	2.44 (1.78)	1.99 (1.39)	< 0.001
LDA	HLSUMTri	7.50 (1.54)	7.49 (1.52)	0.786	2.34 (1.71)	1.94 (1.34)	< 0.001
SVM	HLAVG	7.55 (1.57)	7.47 (1.51)	0.038	2.65 (1.91)	2.02 (1.41)	< 0.001

### Healthcare service utilization

Finally, analyses of healthcare service utilization rates demonstrated that patients with predicted limited HL had on average 10 outpatient clinic visits annually, compared to an average of 8 to 9 among patients with adequate HL. Similar differences were found for emergency room visits (0.53 vs. 0.31) and inpatient hospitalizations (0.25 vs. 0.13; see Table 6). These were significant for all models, although the differences in emergency room visits and inpatient hospitalizations were again most robust for the SVM HLAVG version.

**Table 6:** Healthcare service utilization (outpatient clinic visit, emergency room encounter and hospitalization–Mean (SD)).

ML Algorithm for Literacy Profiles	Literacy Profile (Dependent Variable)	Outpatient clinic visit		ED visits		Hospitalization		P-value
		Limited HL	Adequate HL	Limited HL	Adequate HL	Limited HL	Adequate HL	
LDA	HLCOMB	10.02 (10.4)	8.76 (8.76)	0.46 (1.07)	0.30 (0.75)	0.21 (0.68)	0.13 (0.51)	< 0.001
LDA	HLSUMTri	9.69 (10.0)	8.79 (8.81)	0.42 (1.00)	0.31 (0.75)	0.19 (0.63)	0.14 (0.56)	< 0.001
SVM	HLAVG	10.29 (10.7)	9.01 (9.16)	0.53 (1.20)	0.31 (0.76)	0.25 (0.73)	0.13 (0.54)	< 0.001

## Discussion

The objective of the study was to examine the extent to which limited HL can be identified through the linguistic features of DM patients’ secure messages. We compared three LPs modeled from different derivations of patients’ self-reported HL using multiple ML algorithms and determined the LP that best predicted self-reported HL. The SVM LP model for HLAVG performed quite well with respect to self-reported HL for all the metrics except specificity, and it generated the best balance with respect to PPV and NPV. In addition, HLAVG predicted that about 1/3 of patients have limited HL, consistent with prior research. Finally, with respect to confirmation of previous correlations between accepted measures of HL and health outcomes, the LP derived from the HLAVG SVM model clearly performed the best.

Overall, we found that several linguistic features that measure different language aspects of SMs derived from electronic patient portals yielded models that predicted self-reported HL with a modest but acceptable degree of accuracy. Together, these features, including less sophisticated and less positive language, provide us with a language profile of limited HL patients. While the linguistic features we included have been previously studied to classify literacy [82–83], the texts that have been assessed have not been derived from e-mail messages. We found that combinations of language features can be applied to SMs to successfully discriminate patients based on self-reported metrics of HL. To our knowledge, this represents the first successful attempt to use NLP to identify patients who have higher likelihoods of self-reported limited HL and vulnerability to worse health outcomes.

The ultimate goal of this work is to develop tools to improve communication between clinicians and patients so as to foster “shared meaning”. Measuring HL has traditionally been extremely challenging at both the individual and population levels, given the time and personnel demands intrinsic to current HL measurement approaches. An automated LP could provide an efficient means to help identify the subpopulation of patients with limited HL. Given that limited HL is an important and potentially remediable factor influencing the incidence of, complication rates of, and mortality from DM and other chronic diseases, developing a valid method for rapid HL assessment represents a significant accomplishment with potentially broad public health and clinical benefits. For instance, identifying patients likely to have limited HL could prove useful for alerting physicians about potential difficulties in comprehending written and/or verbal instructions. This lack of comprehension is particularly critical when there are significant drug safety concerns, e.g., anticoagulants and insulin [97]. Additionally, patients identified as having limited HL could be flagged to receive follow up communications to ensure understanding of medication instructions and adherence [98].

## Limitations and future work

Our study has important limitations. First, while our patient sample was large and ethnically diverse, and we studied a large number of patients’ SMs, we were only able to analyze those patients who had engaged in SM with their physicians. As such, the SM-based method used in this study can only be applied to patients who use SM. However, recent data suggest that patients with limited HL are accelerating in their use of patient portals, and at least 2/3 of KPNC diabetes patients with limited HL now use the patient portal. Second, we limited the study to only English SMs, excluded second language patients who may have limited HL. At the time of this study, KPNC did not have a Spanish language portal. Third, our LPs were only modeled against self-reported HL.

Our future research will compare performance of these LP models with novel LPs derived from (a) linguistic expert ratings of SMs, (b) existing and simpler linguistic indices that estimate literacy, and (c) a more limited set of linguistic indices obtained after the ablation test. We plan to examine the relative performance of these LPs in safety net healthcare systems, as well as in patient populations with conditions other than DM. Fourth, while limited HL is more heavily concentrated in safety net healthcare settings; this phase of our research involved a fully insured population (KPNC) because of the availability of extensive linguistic and health-related data. However, KPNC has a sizable Medicaid population, and over 1/3 of their DM patients have limited HL [4, 84]. Moreover, KPNC members are ethnically diverse and largely representative of the U.S. population, with the exception of extremes of income, and working in an integrated system ensures that we had complete capture of medication refills and healthcare utilization. Finally, while our cross-sectional bivariate analyses with respect to health outcomes were confirmatory, future work will utilize longitudinal data to examine whether LPs are independently associated with changes in health.

## Conclusion

Because HL limitations pose a barrier to patient-provider communication, undermine healthcare delivery, and can jeopardize health outcomes, the ability to assess patients’ HL has long been of interest to individual clinicians, healthcare delivery systems, and the public health community. To date, measuring HL so as to tailor interventions to help overcome this vulnerability [98] has proven painstaking and infeasible to scale. Health systems are increasingly incorporating predictive models and derived scores as a means of risk stratifying and targeting care. Using “big data” to estimate HL at the individual patient level could open up new avenues to enhance population management as well as individualized care. Failure to do so in population management interventions has previously been shown to amplify HL-related disparities [99].

Our LPs offer healthcare delivery systems a novel, automated, and economical way to identify the subset of patients who have higher likelihoods of having limited HL. One major advantage of the SM-based LP described in this paper is that it does not require patients to self-report literacy limitations or complete detailed literacy assessments, thus avoiding time-consuming, expensive and intrusive data collection. If the value of the LP we have developed can be replicated in other populations, settings and/or conditions, we believe the LP has the potential to enable HL estimation in a majority of patients, given the rapid expansion of patient portals and associated secure messaging. Our work demonstrates that, for any patient who sends to their care team at least one SM of 50 words or more, health systems can extract linguistic features from these SMs using the NLP tools described above, and employ the machine learning trained model to obtain an LP, thereby categorizing the patient’s HL as adequate or limited. This LP could be used to target and tailor both communication and clinical interventions at the health system level. In addition, LPs could be employed as a provider alert for HL limitations in the EHR to improve individual-level communication, be it in person or via SM. Finally, we are extending our patient-level LP work to develop parallel profiles that measure clinician text complexity. This will (1) create new opportunities to study the prevalence and salutary effects of clinician-patient communication concordance, and (2) enable health systems to provide general feedback and training to clinicians whose communication may be overly complex, or provide specific, automated, real-time feedback to clinicians as they are composing SMs so as to reduce text complexity.

Based on our results, we recommend that researchers and health system planners interested in using NLP to estimate HL use the version of the LP that we have named SVM HLAVG. While the LP is only a proxy measure of barriers to health-related communication, our research demonstrates that LP (SVM HLAVG) is associated with both self-reported HL as well as a broad range of health outcomes previously shown to be sensitive to HL (e.g., medication adherence, A1c, hypoglycemia, comorbidities, and utilization). Our future work will (1) compare alternative methods to estimate HL, including those derived from expert ratings, previously validated more simple linguistic indices, and a more limited set of linguistic indices obtained after an ablation test, (2) develop similar measures for clinicians’ SMs to measure linguistic discordance with patients, (3) determine if automated feedback to clinicians improves SM linguistic concordance, and (4) extend this research to safety net healthcare settings and other conditions. We believe that this innovative tool can facilitate a comprehensive and economical classification of patient HL among those who use SM to communicate with their healthcare provider. Given our method has been validated in one large, integrated health system that cares for an ethnically and socioeconomically diverse population, it is reasonable to carry out implementation research that operationalizes and evaluates this tool in this other healthcare settings, and in other health conditions. conditions.

## Competing interests

We have the following interests: Andrew J. Karter and Jennifer Y. Liu are employed by the non-profit health system, Kaiser Permanente Northern California (KPNC). No funding from the KPNC was used to underwrite the research, although KPNC members (patients) may benefit from this research if it employs the Literacy Profiles developed through this research. While Courtney R. Lyles and Dean Schillinger are Adjunct Faculty of the Kaiser Permanente Northern California Division of Research, they are employed by the University of California San Francisco and receive no funds from KPNC. Danielle S. McNamara owns a company Adaptive Literacy Technologies LLC. However, no funding from the company was used to underwrite the research and the company will not benefit from this research. There are no patents, products in development or marketed products to declare. These competing interests do not alter our adherence to all the PLOS ONE policies on sharing data and materials.

## Supporting information

S1 Table Survey questions coding and definitions. (PDF)
