# Supplementary material for: Using natural language processing and machine learning to classify health literacy from secure messages: The ECLIPPSE study
Source: PLoS One. 2019 Feb 22;14(2):e0212488. doi: 10.1371/journal.pone.0212488 (PMC6386302; doi:10.1371/journal.pone.0212488)
Supplement: S1 Table — (PDF) [file pone.0212488.s001.pdf]

Table S1. Survey questions coding and definitions

Variable	Survey Question	Coding
HL Problems (HLPROB)	How often do you have problems learning about your medical condition because of difficulty understanding written information?	1 = "Always"<br>2 = "Often"<br>3 = "Sometimes"<br>4 = "Rarely"<br>5 = "Never"<br>98 = "Don't know"<br>99 = "Refuse"
HL Help (HLHELP)	How often do you have someone help you read Kaiser health plan materials?	
HL Labels (HLLABELS)	How often have you had problems understanding the labels and instructions on your medication bottles or boxes?	
HL Confidence (HLCONF)	How confident are you filling out medical forms by yourself?	1 = "Extremely"<br>2 = "quite a bit"<br>3 = "Somewhat"<br>4 = "A little"<br>5 = "Not at all"<br>98 = "Don't know"<br>99 = "Refuse"
HL Problems (binary; HLPROB2)	If HLPROB is 5 then HLPROB2 is 0<br>otherwise it is 1	0 (no problems)<br>1 (problems)
HL Confidence (binary; HLCONF2)	If HLCONF is either 1 or 2 then HLCONF2 is 0<br>otherwise it is 1	0 (confident)<br>1 (low confidence)
HL Help (binary; HLHELP2)	If HLHELP is 5 then HLHELP2 is 0<br>otherwise it is 1	0 (never needs help)<br>1 (needs help)
Combined HL (HLCOMB)	If either HLPROB2, HLCONF2, or HLHELP2 is 1 then HLCOMB is 1;<br>Otherwise it is 0;	0/1<br>1 -- means having health literacy limitations
Summation of HL (HLSUM)	Sum of HLPROB, (6- HLCONF), and HLHELP	continuous values<br>higher score → better health literacy
Summation of HL (Binary; HLSUMBi)	If HLSUM lies between 0 and 10 then HLSUMBi is 0<br>otherwise it is 1	0 = inadequate health literacy<br>1 = adequate health literacy
Summation of HL (Trinary; HLSUMTri)	If HLSUM lies between 0 and 10 then HLSUMTri is 0<br>If HLSUM lies between 10 and 15 then HLSUMTri is 1<br>otherwise HLSUMTri is 2	0 = inadequate health literacy<br>1 = marginal health literacy<br>2 = adequate health literacy
HLAVG	Average of HLPROB, (6- HLCONF), HLHELP, and HLLABELS	continuous values<br>HLAVG >3 → better health literacy
